# Fructose-1,6-bisphosphatase 1 (FBP1) is an independent biomarker associated with a favorable prognosis in esophageal adenocarcinoma

**DOI:** 10.1007/s00432-022-04025-x

**Published:** 2022-04-27

**Authors:** Alexander Damanakis, Patrick Sven Plum, Florian Gebauer, Wolfgang Schröder, Reinhard Büttner, Thomas Zander, Christiane Josephine Bruns, Alexander Quaas

**Affiliations:** 1 Department of General, Visceral, Cancer and Transplantation Surgery, Faculty of Medicine and University Hospital Cologne, Cologne, Germany; 2 Institute of Pathology, Faculty of Medicine and University Hospital Cologne, Cologne, Germany; 3 Department I of Internal Medicine, Center for Integrated Oncology (CIO), Faculty of Medicine and University Hospital Cologne, Cologne, Germany; 4 Gastrointestinal Cancer Group Cologne (GCGC), Cologne, Germany

**Keywords:** Esophageal adenocarcinoma, EAC, Fructose-1,6-bisphosphatase 1 (FBP1), Biomarker, Prognosis, Biomarker, Neoadjuvant therapy, Treatment response, Neoadjuvant treatment

## Abstract

**Introduction:**

Despite modern multimodal therapeutic regimens, the prognosis of esophageal adenocarcinoma (EAC) is still poor and there is a lack of biological markers estimating the patients’ prognosis. Fructose-1,6-biphosphatase (FBP1) is a key enzyme in gluconeogenesis and is associated with tumor initiation in several cancers. Therefore, this study aims to characterize its implication for EAC patients.

**Methods and materials:**

A total of 571 EAC patients who underwent multimodal treatment between 1999 and 2017 were analyzed for FBP1 expression using immunohistochemistry.

**Results:**

82.5% of the EACs show FBP1 expression in the tumor albeit with different intensities categorizing specimens accordingly into score 0 (no expression), score 1 (weak expression), score 2 (moderate expression) and score 3 (strong expression) (score 1 = 25.0%, score 2 = 35.9%, score 3 = 21.5%). Intratumoral FBP1 expression was significantly associated with a better prognosis (*p* = 0.024). This observation was particularly relevant among patients who received primary surgery without neoadjuvant treatment (*p* = 0.004). In multivariate analysis, elevated FBP1 expression was an independent biomarker associated with a favorable prognosis.

**Discussion:**

Despite being associated with a favorable prognosis, the majority of patients with high FBP1 expression also require individualized therapy options to ensure long-term survival. Recently, it has been shown that the presence of the FBP1 protein increases the sensitivity of pancreatic cancer cells to the bromodomain and extraterminal domain (BET) inhibitor JQ1.

**Conclusion:**

We described for the first time the prognostic and possibly therapeutic relevance of FBP1 in EAC. The efficiency of the BET inhibitor in EAC should be verified in clinical studies and special attention should be paid to the effects of neoadjuvant therapy on FBP1 expression.

**Supplementary Information:**

The online version contains supplementary material available at 10.1007/s00432-022-04025-x.

## Introduction

The adenocarcinoma of the esophagus (EAC) has been showing an increase in incidence for years, especially in Western countries. EAC is a malignant epithelial tumor with glandular differentiation. Histologically, the tumors are mucin producing and may occasionally show foci of squamous or endocrine differentiation. Rarely, there is the formation of signet ring cells, papillary structures, or Paneth cells. Despite a slight improvement in the overall prognosis since the introduction of neoadjuvant therapy, esophageal adenocarcinoma still only shows a 5 years survival of about 25%. The main risk factor for the development of EAC is Barrett mucosa, which is associated with reflux esophagitis. Male sex and obesity have also been identified as significant risk factors. In recent years, considerable progress has been made in the genomic characterization of EAC. However, individualized therapeutic approaches except for Her2/neu blockade have not been established yet (Lambert and Halnaut 2007; Lepage et al. 2008; Xie et al. 2017; Wheeler and Reed 2012; Kim et al. 2017).

Fructose-1,6-biphosphatase (FBP1) is a key enzyme in gluconeogenesis (Yasuda et al. 2021). FBP1 inhibits katabolic metabolic pathways such as glycolysis guaranteeing tumor cells an ongoing supply of nutrients via lactate or amino acids to feed the biosynthetic pathways (Grasmann et al. 1872). Nevertheless, its role in solid tumors is still ambiguous. In colorectal cancer, inhibition of FBP1 by upregulation of its upstream target FOXC1 results in increased tumor proliferation (Li et al. 2019) caused by metabolic reprogramming of the malignant cells. Similarly, in human hepatocellular carcinoma and colon cancer promoter hypermethylation of FBP1 inhibited the tumor-suppressive effect of this enzyme (Chen et al. 2011). In esophageal cancer, only little is known about the function of FBP1 and its impact on tumor characteristics. In esophageal squamous cell cancer, it has been shown that FBP1 promotes proliferation, migration, and invasion by regulating fatty acid metabolism in in vitro experiments (He et al. 2021).

This study aims to analyze the putative prognostic impact of FBP1 in a large EAC cohort and therefore describe the possibility of this enzyme as a biomarker within this context.

## Methods and materials

### Study cohort and patient’s characteristics

A total of 790 patients underwent surgery (either primary or as part of a multimodality therapy concept) between 1999 and 2017 at the Department of General, Visceral, Cancer and Transplantation Surgery, University Hospital of Cologne, Germany. The standard surgical procedure was laparotomic or laparoscopic gastrolysis and right transthoracic en bloc esophagectomy including two-field lymphadenectomy of mediastinal and abdominal lymph nodes. Reconstruction was performed by high intrathoracic esophagogastrostomy as described previously (Plum et al. 2021; Hölscher et al. 2007). Patients with advanced esophageal cancer (cT1N1M0 or cT2-3N0-1M0) received preoperative chemoradiation (5-FU, cisplatin, 40 Gy as treated analog the CROSS trial) or perioperative chemotherapy following the FLOT regime (Hagen et al. 2012; Al-batran et al. 2008). All patients were followed up according to a standardized protocol. During the first 2 years, patients were followed up clinically in the hospital every 3 months. Afterward, annual examinations were carried out. These follow-up examinations included a detailed history, clinical evaluation, abdominal ultrasound, chest X-ray, and additional diagnostic procedures as required. Follow-up data were available for all patients. Patient characteristics are given in Table 1.

**Table 1:** Patient’s characteristics and FBP1 protein analysis (n = 571)

Patient’s characteristics			Total	FBP1 expression		*p value*
				Negative	Positive	
All patients			571	243	328	
			100.0%	42.6%	57.4%	
Sex	Female	No	70	35	35	0.198
		%	12.3%	50.0%	50.0%	
	Male	No	501	208	293	
		%	87.7%	41.5%	58.5%	
Age group	< 65 years	No	301	138	163	0.120
		%	52.7%	45.7%	54.3%	
	> 65 years	No	270	105	165	
		%	47.3%	39.0%	61.0%	
Tumor stage	pT1/2	No	155	69	86	0.703
		%	27.4%	44.5%	55.5%	
	pT3/4	No	411	174	237	
		%	72.6%	42.3%	57.7%	
Lymph node metastasis	pN0	No	223	87	136	0.192
		%	39.4%	39.0%	61.0%	
	pN +	No	343	154	189	
		%	60.6%	44.9%	55.1%	
UICC stage	I	No	113	43	70	0.201
		%	20.0%	38.1%	61.9%	
	II	No	136	55	81	
		%	24.1%	40.4%	59.6%	
	III	No	248	107	141	
		%	44.0%	43.1%	56.9%	
	IV	No	67	36	31	
		%	11.9%	53.7%	46.3%	
Neoadjuvant therapy	No	No	248	98	150	0.202
		%	43.40%	39.50%	60.50%	
	Yes	No	323	145	178	
		%	56.60%	44.90%	55.10%	

In this table, the patients were divided into two groups. All patients with absent FBP1 (*n* = 100) or weak (*n* = 143) FBP1 expression were combined (group: absent to low) and the patients with moderate (*n* = 205) or strong (*n* = 123) FBP1 expression in the tumor were combined (group: moderate to strong)

### Protein analysis

In our cohort of 571 patients, the protein status of fructose-1,6-bisphosphatase 1 (FBP1) was determined using immunohistochemistry (IHC). We used a tissue microarray (TMA) with formalin-fixed and paraffin-embedded tumor material. For tissue microarray analysis, one tissue core from each tumor was punched out and transferred into a TMA recipient block. Tissue cylinders with a diameter of 1.2 mm each were punched from selected tumor tissue blocks using a self-constructed semi-automated precision instrument and embedded in empty recipient paraffin blocks. Four-micrometer sections of the resulting TMA blocks were transferred to an adhesive-coated slide system (Instrumedics Inc., Hackensack, NJ) for immunohistochemistry. Immunohistochemistry (IHC) was performed on TMA slides using a rabbit monoclonal antibody against FBP1 (Abcam, clone EPR4619, 1:100 with EDTA buffer) with the automated stainer from Leica Bond, Germany. FBP1 expression was detected within the cytoplasm of TMA samples deriving from the resulting surgical specimens after either primary resection or following prior neoadjuvant treatment. Preoperative tissue samples were not included in the analysis. All stains ran in one immunochemical run accompanied by a positive control. We used liver as well as kidney as positive control.

The evaluation was primarily carried out in a four-step scheme as follows: Score 0 = no expression of FBP1; Score 1 (weak expression) = up to 70% of tumor cells show a weak intensity or up to 30% of tumor cells show moderate intensity of staining; Score 2 (moderate expression) = more than 30% of tumor cells show a moderate intensity of staining or more than 70% a weak intensity of staining or up to 30% of tumor cells show a strong intensity of FBP1 expression; Score 3 = strong expression of more than 70% of tumor cells show a moderate intensity of staining or more than 30% of tumor cells show a strong intensity of staining. In a previous publication, we were able to show that this nationally commonly used scoring system correlates excellently with the internationally more common H-score (Moentenich et al. 2020). The H-score is obtained using the following formula: 3 × percentage of strongly stained cells + 2 × percentage of moderately stained cells and percentage of weakly stained tumor cells. The H-score ranges from 0 to 300. An H-score of over 200 corresponds to a score of 3.

Depending on the effect of neoadjuvant chemotherapy or chemoradiation, there is a preponderance of minor responders in the TMAs, defined as histopathological residual tumor of ≥ 10% (Schneider et al. 2008).

### Statistical analysis

Clinical data were collected prospectively and analyzed according to a standardized protocol as previously described (Plum et al. 2021; Plum et al. 2020; Plum et al. 2019). SPSS Statistics for Mac (Version 21, SPSS) was used for statistical analysis. Interdependence between stainings and clinical data were calculated using the Chi-squared and Fisher’s exact tests and displayed by cross-tables. Survival curves were plotted using the Kaplan–Meier method and analyzed using the log-rank test. All tests were two sided. *P* values < 0.05 were considered statistically significant.

## Results

### Patients

All 790 patients who underwent Ivor–Lewis esophagectomy between 1999 and 2017 at our department were included on the TMA. Of these, again only 685 (86.7%) patients were immunohistochemically “interpretable” for FBP1. Reasons for the non-informative cases were missing tissue samples or the absence of distinct cancer tissue in the corresponding TMA spot. Furthermore, there were postoperative follow-up data from 571 of these evaluable patients on the TMA, so ultimately a total of 571 patients who underwent surgery during the specified period qualified for the present study. Overall, 571 patients were included in our analysis, 87.7% were male. More than half of all patients (56.9%) had an advanced tumor stage (UICC stage 3 and stage 4). 56.6% of the analyzed tumor samples were from patients who had received neoadjuvant therapy with either chemotherapy alone (according to FLOT regimen) or combined chemoradiation (according to CROSS protocol). There was no significant difference in UICC stages, sex, lymph node positivity rate, and condition after neoadjuvant therapy in patients with no or weak FBP1 expression and patients with a moderate or strong expression (*p* = 0.201) within the total cohort (see Table 1).

### Protein analysis of fructose-1,6-bisphosphatase 1 (FBP1) and overall survival

**Fig. 1:**
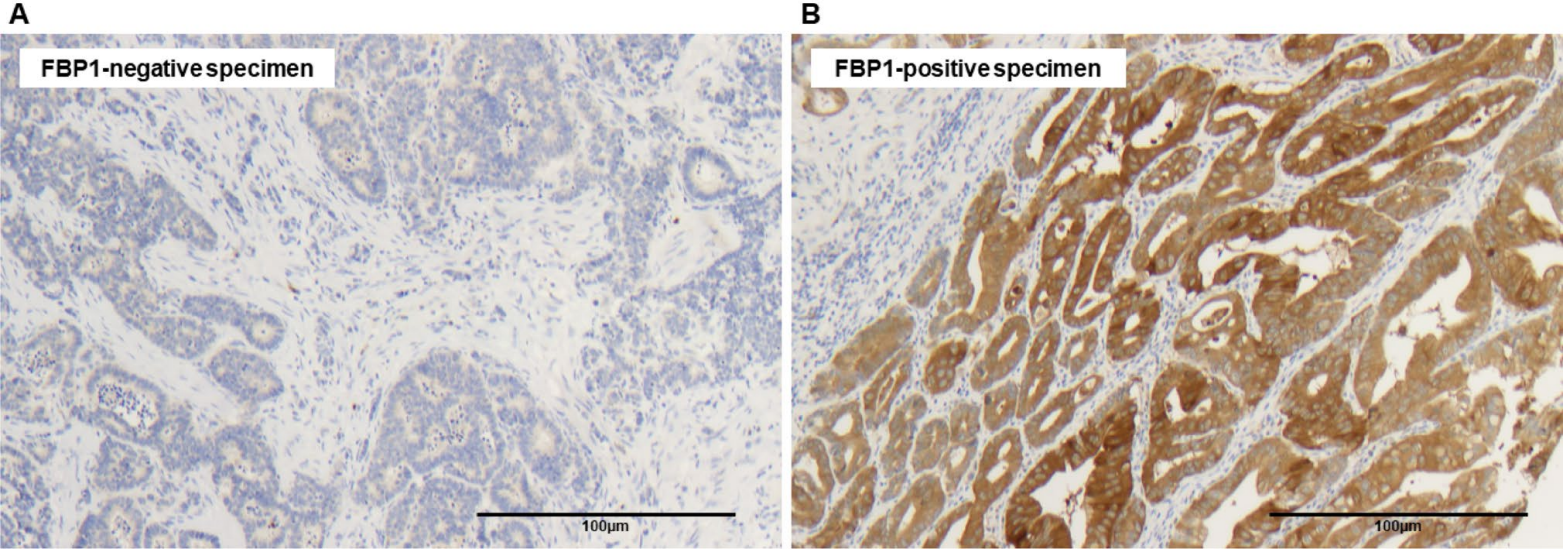
Representative images of immunohistochemistry (IHC) from **A** FBP1-negative (score 0) and **B** FBP1-positive (score 3) specimens of esophageal adenocarcinoma from the tissue microarray. Magnification 100×. Example of a strong FBP1-positive adenocarcinoma (score 3). Homogeneous vigorous expression of FBP1 in the cytoplasm of tumor cells. The surrounding stroma is negative as well as an example of an FBP1 negative EACs

571 patients were examined via immunochemistry for FBP1 expression. 82.5% of them showed FBP1 expression in the tumor albeit with different intensities (score 1 = 25.0%, score 2 = 35.9%, score 3 = 21.5%). Representative images of the immunohistochemical staining are illustrated in Fig. 1.

**Fig. 2:**
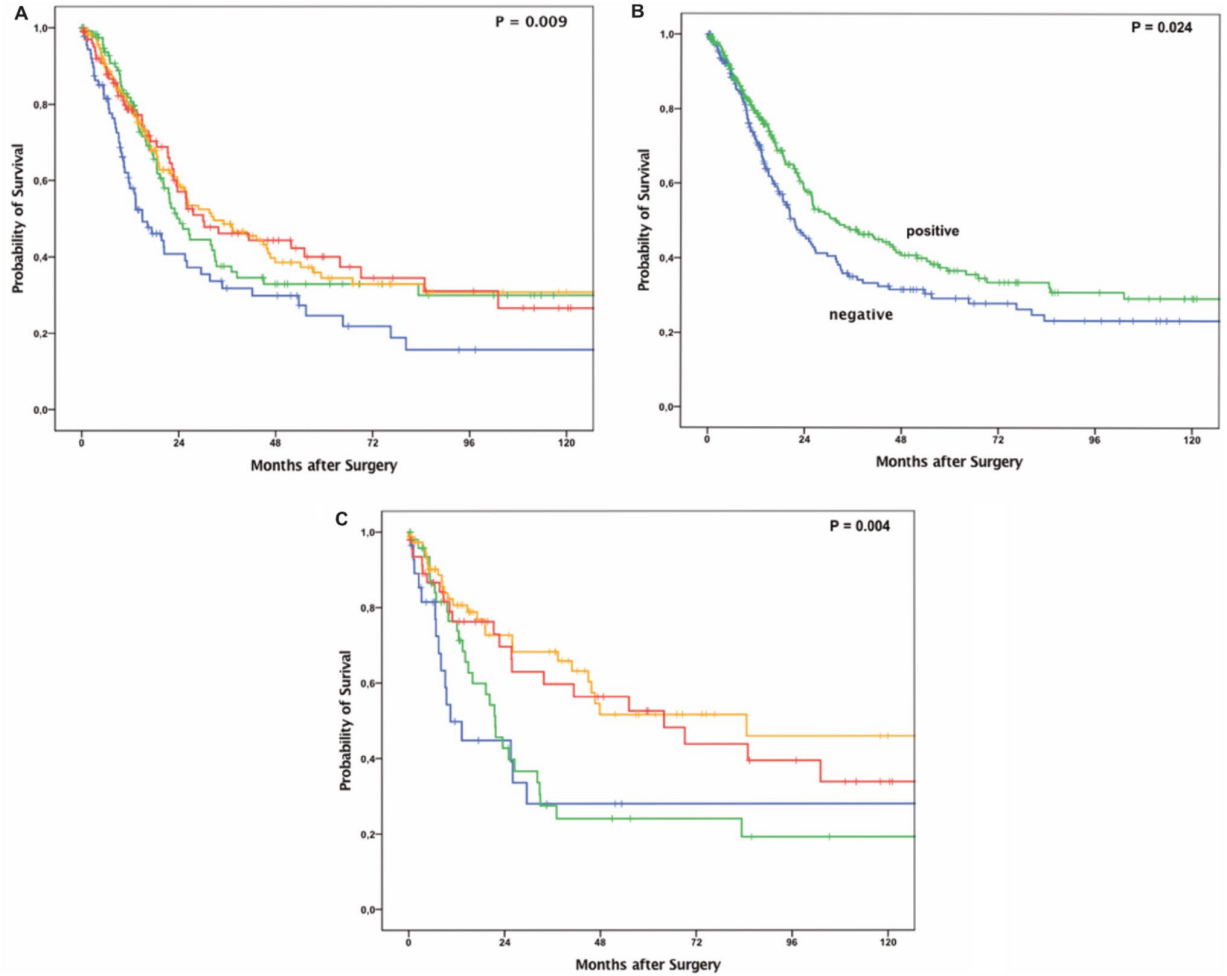
Survival analysis of **A** the entire patient cohort (*n* = 571) according to the different scores of protein expression of FBP1 within the tumor, **B** the entire patient cohort stratified by negative or positive FBP1 expression within the tumor specimens, and **C** FBP1 protein expression in patients with primary surgery (*n* = 248). Patients who had tumors that showed any expression of FBP1 had a better median overall survival. Among patients without neoadjuvant therapy, moderate (red) and strong (orange) FBP1-positive EAC showed a statistically significant better prognosis in comparison to weak positive or negative tumors. Blue = score 0, green = score 1, red = score 2, orange = score 3

The patient characteristics are summarized in Table 1. In the entire patient cohort, any FBP1 expression showed a better overall survival than negative FBP1 (*p* = 0.24, Fig. 2B). We performeded survival analysis of the patients with respect to FBP1 expression (Fig. 2A) revealing median survival times for score 0 = 15.1 months (95% CI 8.26–21.84), score 1 = 24.3 months (95% CI 19.19–29.31), score 2 = 32.7 months (95% CI 18.47–46.85) and score 3 = 30.1 months (95% CI 14.1–46.03), respectively. In the group that underwent primary surgery, patients whose tumors had absent (score 0) and low (score 1) FBP1 expression showed a significantly lower median survival [score 0 = 10.5 months (95% CI 4.45–16.51) and score 1 = 21.6 months (95% CI 16.9–26.40)] than those with medium (score 2) and high FBP1 expression (score 2 = 84.6 (95% CI 9.22–15.98), score 3 = 63.9 months (95% CI 24.62–103.25, *p* = 0.004) (Fig. 2C; Figure S1). This difference was not observed in the group that had received neoadjuvant therapy (Figure S2).

In a multivariate analysis of the entire cohort (taking into account the tumor stages and the nodal stage), FBP1 turned out to be an independent biomarker associated with a favorable prognosis (HR 0.762, 95% CI 0.589, 0.986; *p* value 0.039, Table 2).

**Table 2:** Multivariate analysis revealing positive FBP1 expression associated with statistically significantly lowered HR

	Hazard ratio	95% confidence interval		
		Lower	Upper	*p* value
Sex				
Male vs. female	1.117	0.724	1.723	0.618
Age group				
< 65 vs. > 65 years	1.329	1.027	1.72	0.031
Tumor stage				
pT1/2 vs. pT3/4	1.37	0.973	1.928	0.071
Lymph node metastasis				
pN0 vs. pN +	3.048	2.239	4.148	< 0.001
FBP1 expression				
Negative vs. positive	0.762	0.589	0.986	0.039

### FBP1 and other molecular markers

We also correlated the immunohistochemical expression of FBP1 in EAC specimens with other prognostic markers such as HER2, IDO, VISTA, GATA6, and CD3 that we have previously published (Plum et al. 2020; Plum et al. 2019; Schoemmel et al. 2021; Loeser et al. 2019; Loeser et al. 2020). High FBP1 expression correlated with increased levels of HER2 (*p* = 0.007) as well as IDO (*p* = 0.004) and higher numbers of infiltrating CD3 lymphocytes (*p* = 0.039), while there was no correlation between FBP1 expression and GATA6 (*p* = 0.540) or VISTA levels (*p* = 0.126) (data not shown).

## Discussion

The role of FBP1 in various cancer entities is undergoing scrutiny in the last decade and it is found to be up-regulated in different tumor types (Li et al. 2016a). According to the RNA expression data of TCGA, this is also true for adenocarcinoma of the esophagus (Gao et al. 2013). While FBP1 is ascribed a tumor-inhibiting function in various tumor entities, nothing is known about the role of FBP1 in esophageal adenocarcinoma (Grasmann et al. 1872).

We evaluated the significance of FBP1 expression at the protein level in 571 patients with EAC. We could show for the first time and in a large dataset that FBP1 protein can be identified through immunohistochemistry in the majority of tumors (82% of tumors are FBP1 positive) with different intensities. Our results at the protein level match the RNA expression results of the TCGA data (Figure S3). FBP1 was associated with a favorable prognosis in EAC in our collective of patients. In particular, strongly positive tumors show a favorable disease progression compared to the FBP1 weakly positive or negative tumors. The value of FBP1 as a prognostic marker was underlined in the multivariate analysis of our cohort, where FBP1 was an independent prognostic marker (Table 2). This is in line with the fact that FBP1 is also associated with a better prognosis in other tumor entities supporting the relevance of this cross-entity biological effect. Also, in vitro data in other tumor entities such as prostate, gastric and hepatocellular cancer found FBP1 gene silencing, leading to promotion of epithelial to mesenchymal transition, invasion, and metastasis (He et al. 2021; Li et al. 2016; Li et al. 2020). Data on FBP1’s role in EAC are still scarce, but a recent study in esophageal squamous cell carcinoma also found the loss of FBP1 being associated with increased proliferation, migration, and invasion (He et al. 2021). Interestingly, we identified a positive correlation between higher expression of HER2 (a tyrosine kinase regulating intracellular signal pathways resulting in upregulation of proliferation) and elevated FBP1 levels within our EAC specimens. This is concordant to previous findings describing HER2 amplification to be associated with improved survival in EAC (Plum et al. 2019; Yoon et al. 2012). Similarly, higher FBP1 levels within our EAC cohort were connected to high amounts of CD3-positive T cell infiltration within the tumor a phenomenon for which survival benefit has already been described in this tumor entity (Schoemmel et al. 2021). After all, this data emphasizes the putative prognostic relevance of FBP1 demonstrating its potential as a biomarker (alone or in combination with other molecular markers) for prognostic estimations among EAC patients.

Regardless of its positive prognostic value on overall survival, the majority of patients with high FBP1 still died because of the disease in our cohort. Those patients still need individualized therapy options, as adjuvant therapy regimens available to date seem to be most effective in the short term. Recently, it was shown that the presence of the FBP1 protein increases the sensitivity of pancreatic cancer cells to the bromodomain and extraterminal domain (BET) inhibitor JQ1, highlighting FBP1’s possible role in individualized cancer therapy (Wang et al. 2018). In ovarian cancer, FBP1 was found to regulate proliferation, metastasis, and chemoresistance. Ovarian cancer cell lines with increased FBP1 expression were also sensitized to cisplatin-induced apoptosis (Li et al. 2019). Therefore, adjuvant treatment with a platin-containing regimen could theoretically explain the survival benefit of increased FBP1 expression in our cohort, especially in patients that underwent primary resection without pre-treatment. If proven in further studies, the increased chemosensitivity to platin-based drugs in EAC could help in tailoring individual patient therapies. It is also interesting that FBP1 was found to enhance radiosensitivity in nasopharyngeal carcinoma cells (Zhang et al. 2021). We found the most pronounced survival difference in patients that had not received neoadjuvant treatment. Therefore, according to our data, the role of FBP1’s radiosensitizing effect needs to undergo further study in EAC.

The prognostic relevance of FBP1 was highest in our cohort of patients that underwent surgery without any pre-treatment. Neoadjuvant therapy may influence the FBP1 protein expression measured by immunohistochemistry, an observation that needs to be considered when interpreting the results of FBP1 expression. Especially, if future studies use FBP1 expression to investigate individualized therapies.

## Conclusion

We describe, for the first time, the prognostic and possibly therapeutic relevance of FBP1 in a large cohort of patients with esophageal adenocarcinoma. Treatment options should be further evaluated and possible changes of FBP1 expression through neoadjuvant therapy further investigated.

## Supplementary Information

Below is the link to the electronic supplementary material.

Supplementary file1 (DOCX 281 kb)

## Data Availability

The datasets generated and/or analyzed during this current study are available from the corresponding author on reasonable request.
