# Supplementary material for: Fructose-1,6-bisphosphatase 1 (FBP1) is an independent biomarker associated with a favorable prognosis in esophageal adenocarcinoma
Source: J Cancer Res Clin Oncol. 2022 Apr 27;148(9):2287–93. doi: 10.1007/s00432-022-04025-x (PMC9349078; doi:10.1007/s00432-022-04025-x)
Supplement: Supplementary file 1 — Supplementary file1 (DOCX 281 kb) [file 432_2022_4025_MOESM1_ESM.docx]

**SUPPLEMENTARY FIGURES**


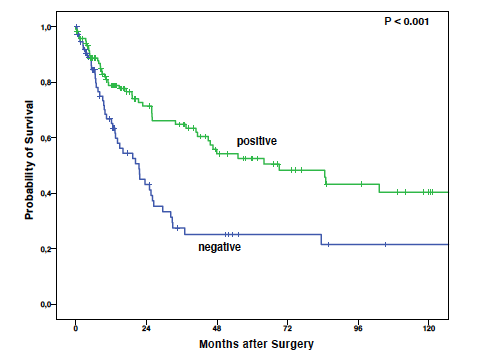


**Figure S1: Median survival in patients that underwent primary surgery stratified by negative or positive FBP1 expression.**


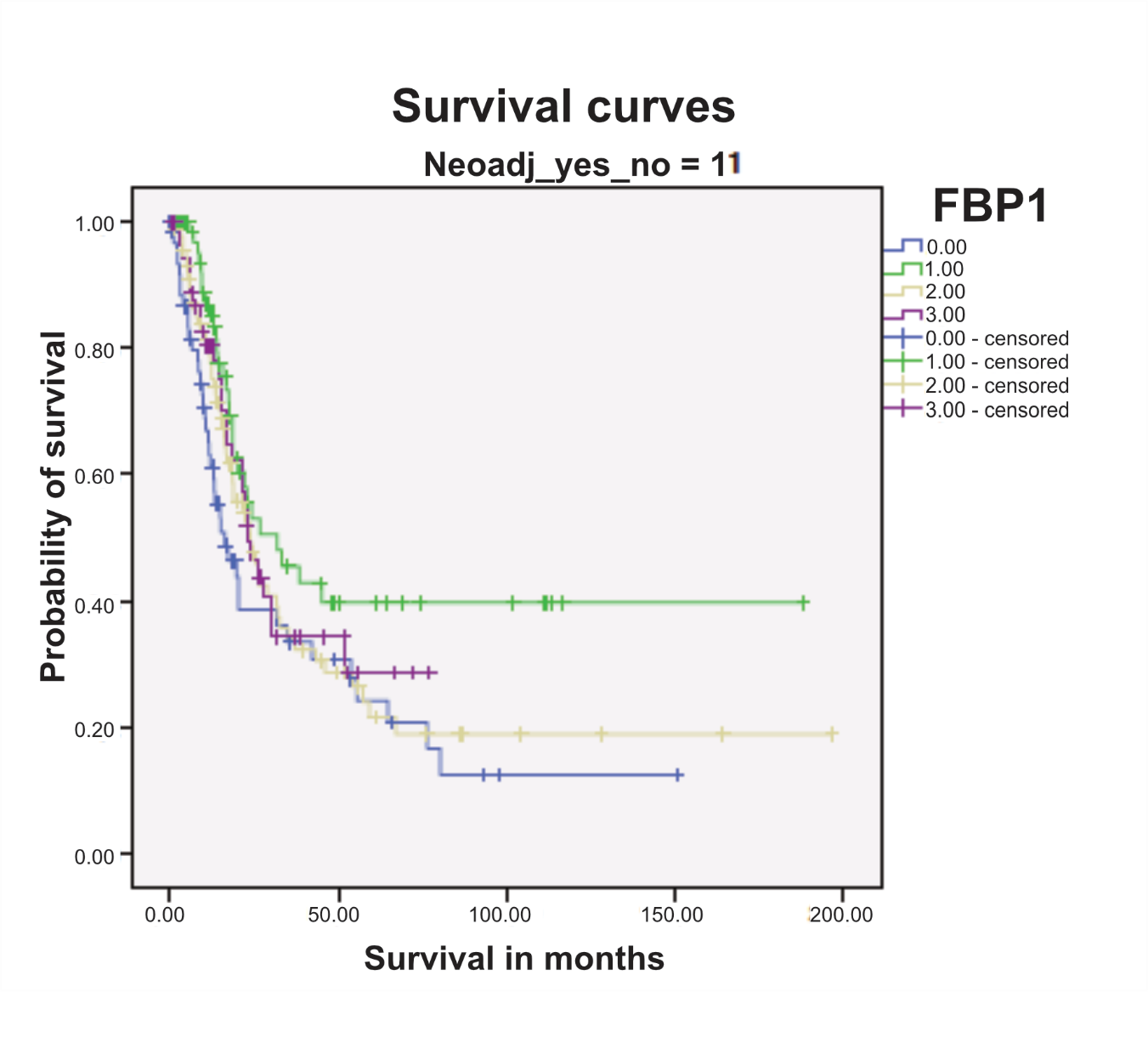


**Figure S2: Median survival according to FBP1 expression in patients that underwent neoadjuvant therapy**


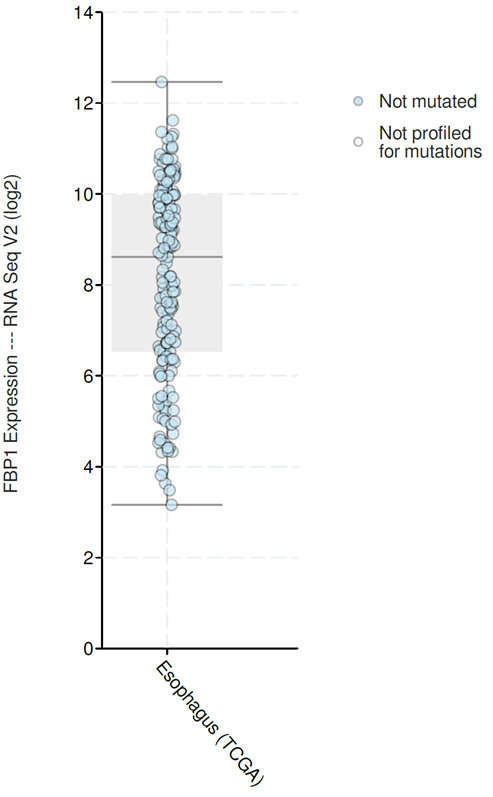


**Figure S3: TCGA data for FBP1 expression in esophageal adenocarcinoma**
